# Early cellular events and potential regulators of cellulase induction in *Penicillium janthinellum* NCIM 1366

**DOI:** 10.1038/s41598-023-32340-x

**Published:** 2023-03-28

**Authors:** Meera Christopher, AthiraRaj Sreeja-Raju, Amith Abraham, Digambar Vitthal Gokhale, Ashok Pandey, Rajeev K. Sukumaran

**Affiliations:** 1 Biofuels and Biorefineries Section, Microbial Processes and Technology Division, CSIR- National Institute for Interdisciplinary Science and Technology, Industrial Estate P.O., Pappanamcode, Thiruvananthapuram, Kerala 695019 India; 2 Academy of Scientific and Innovative Research (AcSIR), Ghaziabad- 201002, India; 3 Department of Chemical Engineering, Hanyang University, Seoul, Republic of Korea; 4 National Chemical Laboratory, Pune, India; 5 Centre for Innovation and Translational Research, CSIR-Indian Institute of Toxicology Research, Lucknow, 226 001 Uttar Pradesh India; 6 Sustainability Cluster, School of Engineering, University of Petroleum and Energy Studies, Dehradun, 248 007 India; 7 Centre for Energy and Environmental Sustainability, Lucknow, 226 029 India

**Keywords:** Fungi, Fungal genes

## Abstract

Cellulase production by fungi is tightly regulated in response to environmental cues, and understanding this mechanism is a key pre-requisite in the efforts to improve cellulase secretion. Based on UniProt descriptions of secreted **C**arbohydrate **A**ctive en**Zymes** (CAZymes), 13 proteins of the cellulase hyper-producer *Penicillium janthinellum* NCIM 1366 (PJ-1366) were annotated as cellulases- 4 cellobiohydrolases (CBH), 7 endoglucanases (EG) and 2 beta glucosidases (BGL). Cellulase, xylanase, BGL and peroxidase activities were higher for cultures grown on a combination of cellulose and wheat bran, while EG was stimulated by disaccharides. Docking studies indicated that the most abundant BGL- Bgl2- has different binding sites for the substrate cellobiose and the product glucose, which helps to alleviate feedback inhibition, probably accounting for the low level of glucose tolerance exhibited. Out of the 758 transcription factors (TFs) differentially expressed on cellulose induction, 13 TFs were identified whose binding site frequencies on the promoter regions of the cellulases positively correlated with their abundance in the secretome. Further, correlation analysis of the transcriptional response of these regulators and TF-binding sites on their promoters indicated that cellulase expression is possibly preceded by up-regulation of 12 TFs and down-regulation of 16 TFs, which cumulatively regulate transcription, translation, nutrient metabolism and stress response.

## Introduction

Lignocellulosic biomass, such as agro-industrial and forestry residues, is an appealing substrate for the production of alternatives to fuels and chemicals derived from fossil fuels. However, commercialization of biomass-to-fuels and chemicals technologies has been hampered by operational and economic constraints, primarily the high cost of cellulases that are used to deconstruct the cellulose. Since industrial cellulases are mostly obtained from fungal sources, a lot of research has been focused on improving the quality and yields of these enzymes.

For the complete mineralization of lignocellulose, fungi employ 3 classes of enzymes- hydrolases, which include cellulases and hemicellulases; lyases and oxidoreductases, which include the lignin degrading enzymes. Cellulose degradation is effected by exoglucanases (EC 3.2.1.91 and EC 3.2.1.74), endoglucanases (EC 3.2.1.4) and betaglucosidases (BGLs) (EC 3.2.1.21). Degradation of the heteropolymeric hemicellulose requires xylan-degrading and side-chain cleaving enzymes. As such, the fungi that can grow on lignocellulose possess a diverse arsenal of enzymes to convert this complex substrate into simpler sugars.

Since the production of extracellular enzymes is an energy-intensive process, the expression of cellulases is triggered only when the organism needs to utilize complex polymers as its energy and carbon source. In the presence of easily metabolizable sources like glucose, cellulase expression is repressed via Carbon Catabolite Repression (CCR)^1^. On encountering a cellulosic substrate, gene expression and protein secretion is triggered by a complex and coordinately regulated network of receptors, transporters, transcription factors (both activators and repressors), and other regulatory proteins.

The mechanisms of fungal cellulase expression have been studied in many organisms, particularly *Trichoderma*, *Aspergillus* and *Neurospora* sp.^2^ However, the complete elucidation of a cellulase production pathway per se has still not been achieved, mainly because the control exercised is highly complex, and also because from genus-to-genus, the mechanism presents both conserved and unique features. Still, certain processes leading to cellulase secretion are believed to be universal among filamentous fungi. For instance, early sensing of the substrate is attained due to the presence of inducers, which are generally oligosaccharides like cellobiose, sophorose, xylose, xylobioses, galactose or lactose. The presence of the inducer in turn activates a downstream signaling cascade which leads to a variety of cellular events- de-regulation of CCR, upregulation of transcriptional activators, re-organization of the chromatin structure and changes in the protein trafficking machinery, all of which culminate in the reorientation of the fungal metabolism to secrete large titers of biomass hydrolyzing enzymes^3^.

Current efforts to bring to life the concept of an “integrated biorefinery”, in which fungi are required to be grown on the lignocellulosic feedstock for production of the necessary enzymes, call for the use of organisms that can secrete high titers of efficient enzymes. In this regard, understanding the mechanisms of cellulase gene regulation will provide vital clues for the systemic improvement of their cellulase production. In this study, we have used *Penicillium janthinellum* NCIM 1366 (PJ-1366)- a strain which has already been reported to have superior cellulase secretion than the gold-standard of industrial cellulase production- *Trichoderma reesei* RUT-C30^4,5^. Since information on cellulase regulation in this genus is limited, we have analysed both the transcriptional and secretome response to induction, and have correlated this data with the distribution of transcription factor (TF) binding sites on the promoters of its cellulases so as to derive an understanding of the regulators and pathways that govern the early induction of cellulases.

## Results

### Time scale analysis of the effect of various carbon sources on protein secretion and cellulase enzyme activities

Fourteen substrates were studied for their effect on enzyme production- the monomers arabinose, glucose and xylose; the disaccharides cellobiose, lactose, maltose and mannose; lignin and its components gluconic acid and vanillin; the polysaccharides cellulose and xylan; wheat bran as a representative of a lignocellulosic substrate, and a combination of wheat bran and cellulose (CW) in the ratio 2.5:1 (w/w) (optimized in previous studies^6^). Seven parameters were monitored- total protein secretion, cellulase activity (**F**ilter **P**aper assay- FPase), endoglucanase activity (**C**arboxy**m**ethyl **C**ellulose assay- CMCase), BGL activity (**p**ara **n**itro**p**henyl β-D-**g**lucopyranoside assay- pNPGase), xylanase activity, peroxidase activity and protease activity. Due to the insoluble nature of some of the substrates, a critical parameter- weight of the fungal biomass- was not analysed.

**Figure 1:**
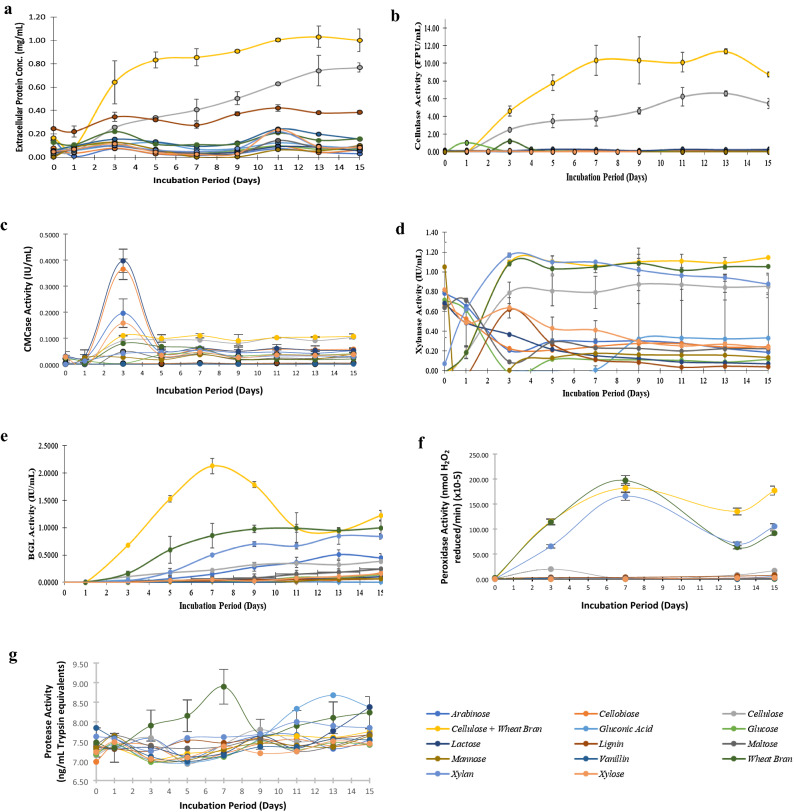
Secreted protein and enzyme activity profiles of PJ-1366 grown on different carbon sources. (**a)** Total protein secretion; (**b)** Total cellulase activity; (**c)** CMCase activity; (**d)** Xylanase activity; (**e)** BGL activity; (**f)** Peroxidase activity, and (**g)** Protease activity *(n* = *3, the error bars indicate standard deviation of mean).*

As seen in Fig. 1, the best inducer of protein secretion and lignocellulase activities was the combination of wheat bran and cellulose, and the protein secretion, FPase and xylanase activities peaked at 3–7 days of incubation, and remained almost steady thereafter. However, BGL and peroxidase activities were maximum at 7^th^ day, and decreased steadily thereafter. From the protease activity profile, it was also seen that adding cellulose to wheat bran led to lower protease activity than when using wheat bran alone.

Contrary to other cellulase activities, endoglucanase activities peaked drastically at the 3^rd^ day of incubation, and was stimulated by disaccharides like cellobiose and lactose, and to a lesser extent by the monosaccharides arabinose and xylose. Wheat bran did not induce much endoglucanase activity, though cellulose, and the cellulose + wheat bran (CW) combination induced similar and steady levels of activity.

### Secretome profiles of induced PJ-1366 cultures

PJ-1366 encodes 11,828 proteins, of which 1007 have signal peptides^7^. Of the extracellular proteins, 216 proteins were described as carbohydrate-active enzymes (CAZymes), of which 165 were Glycoside Hydrolases (GH), that include the cellulases and hemicellulases^8^. Among them, 40 sequences were identified as belonging to putative cellulase families GH2, GH3, GH30, GH5, GH6, GH7, GH12, GH45 and GH131.

To identify the cellulase proteins that are expressed on induction, secretome profiles of cellulose, CW, lactose and wheat bran cultures were analyzed at different time points, i.e., 3, 7 and 13 days post induction. Based on UniProt annotation of the secreted proteins, 13 cellulases- 4 cellobiohydrolases, 7 endoglucanases and 2 BGLs, were detected in the secretome (Table 1). In the case of both cellulose and CW cultures, these cellulases represented 40–48% of the total secretome.

**Table 1:** Secreted cellulases of PJ-1366.

Protein ID	Gene ID	Accession	Description	% Identity	CAZy Family
Cbh1	ctg7180000009921.g242	AGW24292.1	cellobiohydrolase 2 [*P. oxalicum*]	80.622	GH7
Cbh2	ctg7180000014196.g218	CRL19618.1	1, 4-β cellobiohydrolase [*P. camemberti*]	81.704	GH6
Cbh3	ctg7180000014411.g234	ADX86895.1	cellobiohydrolase II [*P. decumbens*]	78.891	GH6
Cbh4	ctg7180000015101.g188	AGW24291.1	cellobiohydrolase 1 [*P. oxalicum*]	83.664	GH7
Eg1	ctg7180000014395.g40	AFG25592.1	endoglucanase Cel5C [*P. decumbens*]	80.183	GH5
Eg2	ctg7180000014428.g38	GAQ03870.1	endoglucanase 7a [*A. lentulus*]	77.755	GH7
Eg3	ctg7180000014484.g138	ABY28340.1	endoglucanase II [*P. decumbens*]	78.832	GH5
Eg4	ctg7180000015246.g308	ACB06750.1	endo-1,4-β-D-glucanase [*P. brasilianum*]	82.324	GH5
Eg5	ctg7180000009921.g262	OKP11846.1	Endoglucanase-5 [*P. subrubescens*]	70.28%	GH45
Eg6	ctg7180000010032.g121	OOQ89941.1	Endoglucanase-1 [*P. brasilianum*]	91.14%	GH12
Eg7	ctg7180000015255.g313	GAT20173.1	Carboxymethylcellulase B [*A. luchuensis*]	46.49%	
Bgl1	ctg7180000014236.g84	CEJ60129.1	Putative Β-glucosidase [*P. brasilianum*]	91.388	GH3
Bgl2	ctg7180000014474.g297	ACD86466.1	β-glucosidase [*P. decumbens*]	88.167	GH3

**Figure 2:**
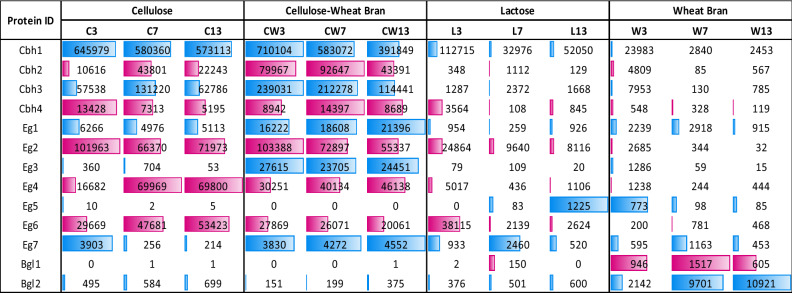
Protein abundance at 3, 7 and 13 days, of PJ-1366 cultures grown on different substrates. The relative abundance of each protein in different substrates is shown by the length of bars.

The most abundant cellulases in the secretome were Cbh1, Cbh3 and Eg2; also, these proteins were highly expressed in the presence of cellulose and moderately induced by lactose (Fig. 2). Wheat bran did stimulate expression of exo- and endoglucanases, and, in agreement with the enzyme activity profiles, the expression could not be sustained. In combination with cellulose, there is a marked increase in the abundance of Cbh2, Cbh3, Eg1 and Eg3. Also, while most of the proteins exhibited a decreasing trend from 3 to 13 days, the endoglucanases Eg1, Eg4 and Eg7 steadily increased in abundance as time passed, which possibly contributes to the overall increase in cellulase (FPase) activity observed in CW cultures (Fig. 1b).

The proteins that were highly expressed in lactose medium were Cbh1, Cbh4, Eg2 and Eg6. Among these, Eg6 is the only protein that has a relatively higher abundance on the 3^rd^ day, and so might result in the peak in CMCase activity observed on the 3^rd^ day of lactose induction (Fig. 1c). Surprisingly, both the BGLs had highest abundance in wheat bran medium and had relatively lower levels in the presence of di- or polysaccharides. Therefore, it is speculated that the higher BGL activity observed in CW medium could be a cumulative effect of the other cellulases. A look at the domains on the endoglucanases of PJ-1366 (Supplementary File 1) shows that Eg1, Eg3, Eg4 and Eg7 contain BGL domains, and from Fig. 2 it can be seen that these proteins are significantly more abundant in the CW medium compared to the other substrates. In fact, EGs with BGL domains have been reported earlier in bacteria^9,10^, with cellobiose-binding EGs predicted to be more thermostable^11^, and a much earlier study by Ladisch et al.^12^ had also reported the cellobiase activity of *T. reesei* endoglucanase. Also, Bgl1 had only negligible abundance in cellulose, CW and lactose, making Bgl2 the major source of cellobiase activity in the cultures.

The cellulolytic machinery of *T. reesei* includes 9 proteins- 2 cellobiohydrolases, 5 endoglucanases and 2 BGLs^13^. The level of BGL activity is critical in determining the efficiency of hydrolysis. Since BGLs function in converting cellobiose to glucose, low BGL activity leads to the accumulation of cellobiose, which in turn represses the action of endoglucanases and exoglucanases. The cellulase complex of *N. crassa* is also low on BGL- comprising of 3 exoglucanases, 4 endoglucanases and 1 BGL^14^. On the other hand, enzyme assays and comparative genome analysis have shown that many *Penicilli* possess more BGL genes and exhibit higher BGL activity than *T. reesei*, which in turn helps to achieve faster and more efficient biomass hydrolysis^15^. In the case of PJ-1366, studies by Sreeja-Raju et al.^4^ has shown that there is no accumulation of cellobiose during biomass hydrolysis, indicating that Bgl2 probably has very high turnover.

### Physicochemical properties of PJ-1366 cellulases

The molecular weights of the cellulases vary from 25 kDa (Eg6) to 93 kDa (Bgl1) (Table 2). Domain analysis of the cellulases (Supplementary File 1) showed that the BGLs contain more expansive GH domains, often incorporating an additional BglX (domain found in periplasmic BGLs) or Fn3-like domain. Fn3 (Fibronectin III) domains primarily function in mediating protein–protein interactions, and collocates in enzyme structure to act as a linker for protein structure stability and activity^16^. In bacteria, Fn3 is found only in extracellular GHs, and is responsible for loosening cellulose surfaces, peeling cellulose fibers and directing cellulose chain into the catalytic core for easy conversion of substrates^17,18^.

**Table 2:** Predicted properties of PJ-1366 cellulases.

Protein	Residues	Mol. Wt. (kDa)	pI	GRAVY score
*Cbh1*	544	56.89	pH 4.71	− 0.308
*Cbh2*	399	41.84	pH 4.55	− 0.082
*Cbh3*	467	48.89	pH 4.89	− 0.048
*Cbh4*	452	47.65	pH 4.25	− 0.289
*Eg1*	656	69.95	pH 5.21	− 0.030
*Eg2*	483	50.23	pH 4.77	− 0.296
*Eg3*	408	43.84	pH 5.51	− 0.156
*Eg4*	608	65.32	pH 5.40	− 0.132
*Eg5*	287	29.74	pH 7.68	− 0.165
*Eg6*	237	25.46	pH 4.67	− 0.108
*Eg7*	510	55.92	pH 6.96	− 0.257
*Bgl1*	778	84.17	pH 5.73	− 0.104
*Bgl2*	862	93.30	pH 4.67	− 0.320

Computations of the Grand Average of Hydropathicity (GRAVY) index predicted that all the cellulases were hydrophilic (negative scores) rather than hydrophobic; this may also be interpreted to mean that these proteins are water-soluble and globular, not fibrous or membrane-bound. pI values ranged from 4.25 to 7.00, however, it may be noted that cellulases tend to be modified post-translationally, especially via glycosylation and acetylation^19^, and such modifications can cause shifts in the pI.

### Activity of PJ-1366 BGLs in the presence of glucose

A common feature of most naturally occurring BGLs is product inhibition by glucose. This feedback loop allows control over unnecessary hydrolysis of substrate, and the synthesis of cellulose hydrolyzing enzymes through the linked repression mechanisms, when there is sufficient glucose. However, for industrial purposes, this feature presents a drawback since it will necessitate higher enzyme addition to attain higher glucose concentrations. Therefore, having BGLs that can function even in the presence of glucose offers an advantage for industrial applications.

**Figure 3:**
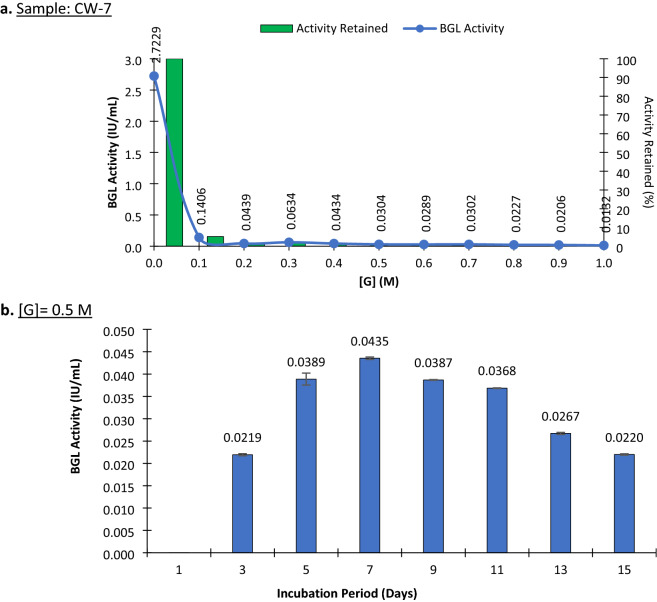
Activity of PJ-1366 BGLs in the presence of glucose. (**a)** BGL activity of CW-7 culture at different glucose concentrations; (**b)** BGL activity of CW cultures at different incubation periods, in the presence of 0.5 M glucose. *(n* = *3, the error bars indicate standard deviation of mean).*

Generally, glucose concentrations in hydrolysis reactors vary from 60 to 90 g/L (0.3–0.5 M). In the case of PJ-1366 BGLs (from CW culture), it was seen that 95% of activity was lost in the presence of 0.1 M glucose, and it reduced to 0.03 IU/mL in 0.5 M glucose, and further to 0.01 IU/mL in 1 M glucose (Fig. 3a). However, previous studies from our lab using a glucose-tolerant BGL of *Aspergillus unguis* have shown that even minute levels of this glucose-tolerant activity is sufficient to bring about drastic improvements in the efficiency of hydrolysis^20^. Keeping with the trend in total BGL production, the glucose-tolerant BGL activity was also maximum at 7th day of incubation (Fig. 3b).

Further, docking studies of PJ-1366 BGLs with the substrate cellobiose, the product glucose, and a potential inducer gentiobiose^21–24^ showed that Bgl1 does not bind glucose in the same site as cellobiose, and Bgl2 has different preferential binding sites for the substrate/inducer and the product (Supplementary File 2). Yang et al.^25^ had demonstrated that residues in the substrate channel are responsible for regulating the relative binding affinity of these sites to glucose. In the case of PJ-1366 Bgl1, both cellobiose and gentiobiose can bind within the substrate channel, while Bgl2 binds only cellobiose in this groove. The binding affinity for glucose in the substrate channel was low compared to cellobiose for Bgl2 (Supplementary File 2), indicating that the structural features of PJ-1366 BGLs may help to alleviate competitive inhibition by glucose.

### Transcriptional response of PJ-1366 cellulases on induction

To further understand the expression of the cellulase genes on induction, transcriptome profiles of glucose (control) and cellulose-grown (test) cultures were analysed. Quantification of transcript levels at 4 h post induction showed that 11 of the 13 cellulases detected in the secretome were upregulated in the presence of cellulose. Of these, the highest expression was of *cbh1*, while the highest fold change in expression was observed for *cbh4*. All 4 of the cellobiohydrolases and 5 of the endoglucanases exhibited > 100-fold increase in transcript levels on induction, while *eg7* and *bgl1* showed a slower response to induction. Also, all the detected transcripts had low levels of expression even in the presence of glucose (Table 3).

**Table 3:**
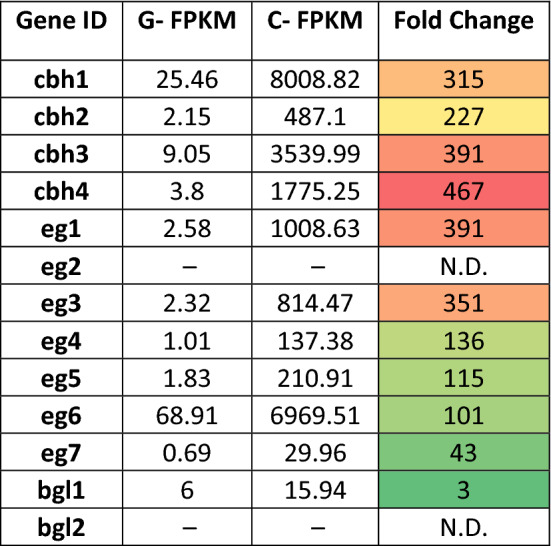
Difference in transcript levels of cellulases at 4 h post induction with cellulose.

G-FPKM represents transcript levels of control (glucose) cultures and C-FPKM is the transcript level in test (cellulose) cultures. N.D.- Not Detected

### Overview of TFs and pathways regulating cellulase secretion in PJ-1366

Transcription factors of PJ-1366 were identified using the Fungal Transcription Factor Database^26^. The database contained 798 TFs from *Penicillium chrysogenum*, and 713 TFs from *Penicillium marneffei*, which matched to 805 PJ-1366 proteins. Mapping of these predicted TFs to the transcriptome detected 758 unique transcripts, of which 369 TFs had a fold change (FC) > 1, and 335 TFs were downregulated on cellulose induction (FC < -1) (Supplementary File 3). 44 TFs were uniquely expressed in the presence of cellulose, while 24 TFs were absent on induction.

Among the TFs uniquely expressed on cellulose, the greatest fold change was observed for a sugar transporter. Domain analysis of the protein showed that it contains both xylE superfamily (xylose transporter) and Gal4 domains (Supplementary File 4). Other TFs with high expression included 3 hypothetical proteins of unknown function.

Ontology analysis using the KEGG server mapped 50% of the TFs to different metabolic and cellular pathways. It was seen that a majority of the differentially expressed TFs were stress-responsive proteins that modulate chromatin structure, signal transduction and protein trafficking within the cell (Table 4).

**Table 4:** Description of PJ-1366 TFs expressed uniquely on cellulose induction.

Gene Id	Transcript ID	FC	Protein	Pathway
ctg7180000015228.g98	TRINITY_DN9813_c0_g1_i2	6.51	Sugar transporter STL1	
ctg7180000015153.g108	TRINITY_DN10034_c0_g1_i1	6.05	Zinc finger, RING-type	
ctg7180000014396.g95	TRINITY_DN25918_c0_g1_i1	4.13	hypothetical protein F1880_008762	
ctg7180000014404.g21	TRINITY_DN20680_c0_g1_i1	4.02	Putative C- × 8-C- × 5-C- × 3-H type zinc finger protein	K00884: Amino sugar and nucleotide sugar metabolism
ctg7180000015063.g244	TRINITY_DN17941_c0_g1_i1	2.81	Protein RDR1	K19815: HOT1; high-osmolarity-induced transcription protein 1
ctg7180000014390.g234	TRINITY_DN10039_c0_g2_i2	2.65	putative transcription factor kapC	K09052: cAMP response element modulator
ctg7180000015095.g290	TRINITY_DN5665_c0_g3_i1	2.35	Putative Zn cluster transcription factor	
ctg7180000014493.g226	TRINITY_DN6170_c0_g1_i1	2.21	Ankyrin repeat domain-containing protein 50	
ctg7180000015091.g54	TRINITY_DN2248_c0_g1_i1	2.16	PGC-1 family protein	
ctg7180000014387.g78	TRINITY_DN51_c0_g2_i1	2.15	Transcription factor gsfR2	K01613: Glycerophospholipid metabolism
ctg7180000015225.g226	TRINITY_DN2774_c0_g3_i1	2.13	Zn2Cys6 transcription factor	
ctg7180000009927.g38	TRINITY_DN12542_c0_g1_i1	1.95	C2H2 finger domain transcription factor CON7	K21455: transcription factor CON7, Master Regulator of Morphogenesis
ctg7180000014395.g39	TRINITY_DN14974_c0_g1_i1	1.75	putative anti-sigma factor rshA	K21989: TMEM63, CSC1; calcium permeable stress-gated cation channel
ctg7180000015231.g282	TRINITY_DN6700_c0_g1_i2	1.72	Uncharacterized transcriptional regulatory protein C417.09c	
ctg7180000009965.g24	TRINITY_DN2504_c0_g1_i1	1.71	Acetamidase regulatory protein	
ctg7180000009917.g189	TRINITY_DN16883_c0_g1_i1	1.44	Fungal transcriptional regulatory protein, N-terminal	
ctg7180000014566.g301	TRINITY_DN4063_c0_g2_i1	1.23	Histone deacetylase complex subunit CTI6	
ctg7180000009913.g131	TRINITY_DN10015_c3_g4_i2	0.54	tRNA dimethylallyltransferase, mitochondrial	K00791: Biosynthesis of secondary metabolites
ctg7180000014518.g184	TRINITY_DN19878_c0_g1_i1	0.46	ER lumen protein-retaining receptor	K23703
ctg7180000014453.g276	TRINITY_DN8767_c1_g1_i1	0.42	C6 zinc finger domain protein	
ctg7180000014524.g251	TRINITY_DN21660_c0_g1_i1	0.23	SCF ubiquitin ligase, Rbx1 component [Posttranslational modification, protein turnover, chaperones]	K03868: RBX1, ROC1; Protein processing in endoplasmic reticulum
ctg7180000014409.g196	TRINITY_DN19037_c0_g1_i1	0.08	FKBP12-associated protein 1	K12236: transcriptional repressor NF-X1
ctg7180000015238.g270	TRINITY_DN4532_c0_g1_i1	0.08	transcription-associated protein; Serine/threonine protein phosphatase 2A, regulatory subunit [Signal transduction mechanisms]	K11584: PPP2R5; mRNA surveillance pathway
ctg7180000014527.g266	TRINITY_DN21736_c0_g1_i1	0.07	Activator of stress 1; Telomere regulation Stn1-like protein	
ctg7180000014579.g109	TRINITY_DN24573_c0_g1_i1	0.07	Putative Transcription factor cys6	
ctg7180000015276.g175	TRINITY_DN16661_c0_g1_i1	0.07	glycosyltransferase family 31 protein	K16165: Amino acid metabolism
ctg7180000015365.g2	TRINITY_DN3342_c0_g2_i1	0.06	TPA: Putative Zn(II)2Cys6 transcription factor	K13354: solute carrier family 25 (peroxisomal adenine nucleotide transporter)
ctg7180000014544.g123	TRINITY_DN13067_c0_g1_i1	0.05	Glutathione-dependent formaldehyde-activating enzyme/centromere protein V	
ctg7180000014391.g271	TRINITY_DN2632_c0_g2_i1	0.04	transcriptional regulator, putative; Mitochondrial sulfhydryl oxidase involved in the biogenesis of cytosolic Fe/S proteins	
ctg7180000014488.g164	TRINITY_DN24494_c0_g1_i1	0.04	F-box/WD repeat-containing protein 7	K03361: CDC4; Ubiquitin mediated proteolysis
ctg7180000014491.g203	TRINITY_DN1805_c0_g2_i1	0.04	PWWP domain protein, Transcription coactivator	
ctg7180000014565.g288	TRINITY_DN7528_c0_g1_i1	0.04	transcriptional corepressor Cyc8	K06665: SSN6, CYC8; general transcriptional corepressor CYC8
ctg7180000014395.g35	TRINITY_DN18970_c0_g1_i1	0.03	Putative Cell differentiation and development protein Fsr1/Pro11	K17608: MAPK signaling pathway (STRN1_3_4; striatin 1/3/4)
ctg7180000014413.g304	TRINITY_DN7855_c0_g3_i1	0.03	Retrograde regulation protein 2	K11369: RTG2; Histone modification proteins
ctg7180000015069.g290	TRINITY_DN18874_c0_g1_i1	0.03	Predicted E3 ubiquitin ligase	
ctg7180000009996.g263	TRINITY_DN7138_c2_g1_i1	0.02	Zn(II)2Cys6 transcription factor	
ctg7180000009999.g272	TRINITY_DN15933_c1_g1_i1	0.02	Regulatory protein wetA	
ctg7180000014898.g253	TRINITY_DN376_c0_g2_i1	0.02	Nitrogen assimilation transcription factor nirA	K13220: Spliceosome associated proteins
ctg7180000015109.g66	TRINITY_DN13365_c0_g1_i3	0.02	Ribosome assembly protein 4	K14855: RSA4, NLE1; Ribosome biogenesis
ctg7180000015224.g183	TRINITY_DN2800_c0_g2_i1	0.02	putative arsenite resistance protein Ars2	
ctg7180000015252.g213	TRINITY_DN2004_c0_g3_i1	0.02	Sterol uptake control protein	K00485: Xenobiotics biodegradation and metabolism
ctg7180000015276.g177	TRINITY_DN12933_c0_g1_i1	0.02	Quinate permease	K14258: TRET1; facilitated trehalose transporter
ctg7180000015378.g133	TRINITY_DN7645_c0_g1_i2	0.02	Ypt/Rab-specific GTPase-activating protein GYP1	K20360: Endoplasmic reticulum (ER)—Golgi transport
ctg7180000014393.g347	TRINITY_DN9938_c0_g3_i1	0.01	F-box and WD domain protein; Beta-TrCP (transducin repeats containing)/Slimb proteins	

In order to uncover potential regulators of the cellulases, the upstream regions of the ORFs (up to 1500 bp) were analysed for the presence of binding sites of known fungal TFs. A total of 51 TFs were selected which can potentially bind on the promoter regions. Ontology analysis of these factors mapped them to a variety of biological processes, i.e., reproduction (GO:0000003), response to stimulus (GO:0050896), cellular process (GO:0,009,987), metabolic process (GO:0008152), biological regulation (GO:0065007) and multi-organism process (GO:0051704) (Supplementary File 5).

**Figure 4:**
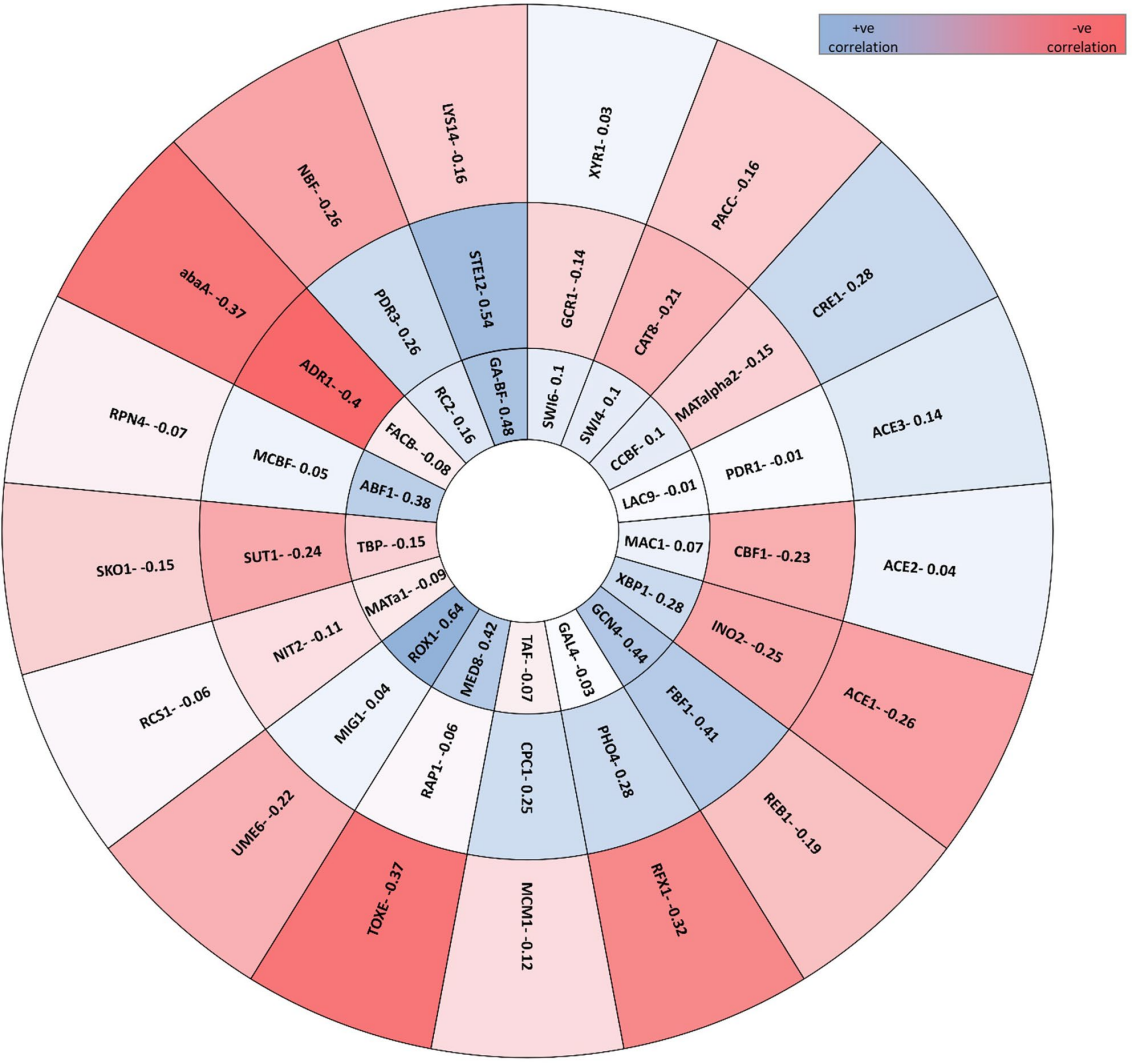
Correlations in protein abundance vs distribution of TF-binding sites in their respective promoters. Blue indicates positive correlation between the occurrence of TF binding sites on the promoter of each cellulase with their abundance in the secretome, and red shows negative correlation. Correlation between TFs for not considered.

The secretome response (protein abundance) of 7 day old cultures grown on cellulose-wheat bran was correlated with the distribution of the number of binding sites for each TF. Binding site frequency was found to be positively correlated with protein abundance for 22 factors, among which the highest positive correlation was observed for Rox1 (0.64), Ste12 (0.54) and Gcn4 (0.44) (Fig. 4). Of these, protein sequence data was available for 16 factors. Using BLASTP analysis, it was seen that PJ-1366 contained homologs for 15 factors. Further, the amino acid metabolism regulators Gcn4 and Cpc1 aligned with the same protein, as did Mig1 and Cre1 making the total number of homologs 13 (Table 5).

**Table 5:**
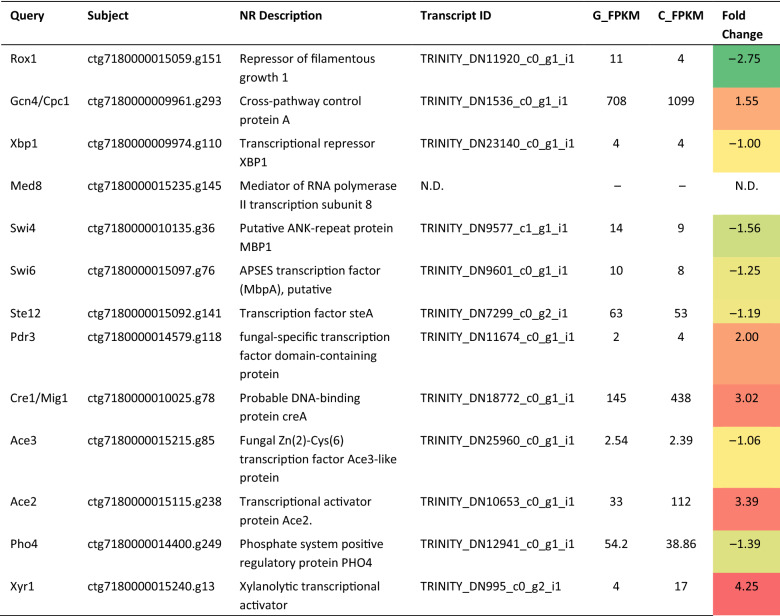
Description of the PJ-1366 homologs of regulators of cellulase expression, and mapping to the transcriptome.

Mapping of these 13 proteins to the transcriptome showed that Xyr1 had the highest differential expression on cellulose induction, although the highest number of transcripts were detected for Gcn4 (Table 5). Rox1, which is a repressor protein, was the most downregulated, as were the Swi4-Swi6 complex, and Ste12. No transcript was detected for Med8, a protein that is known to exercise both positive and negative control on transcription, and no change in expression was seen for Xbp1, another transcriptional repressor. On the whole, it was seen that in addition to known cellulase regulators like Cre1, Xyr1 and the Ace proteins, expression of cellulases also seems to be regulated by factors participating in transcriptional control (Rox1, Xbp1, Med8, Pdr3), growth and cell cycle regulation (Rox1, Swi4-Swi-6), control of phosphate metabolism (Pho4), and global regulators like Gcn4 and Ste12.

**Figure 5:**
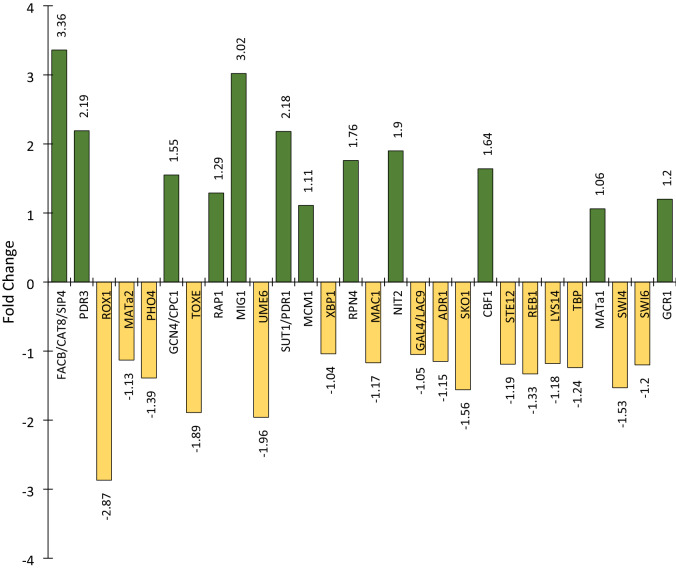
Fold change in expression of proteins predicted as early regulators of cellulase gene expression.

The promoters of these 13 TFs, in turn, contained binding sites for a further 45 transcription factors. Of these, 7 factors- Rc2, Taf, GA-BF, CCBF, Fbf1, Nbf, and Mcbf do not have any identified protein sequences in the TRANSFAC database. Out of the remaining 38 factors, 35 aligned with 30 PJ proteins (there were no homologs in PJ-1366 for Abf1, Rcs1 and Ino2). FacB, Sip4 and Cat8 aligned with the same protein; Gcn4 and Cpc1 were the same protein; Sut1 and Pdr1 aligned with the same protein; and Gal4 and Lac9 were the same protein leaving 29 transcription factors. Of these 29 factors 28 matched to the glucose and cellulose transcriptomes and Med8 did not have a match in the transcriptome. Among the 28 transcripts, abaA was not differentially expressed (Supplementary File 6). The difference in expression of the remaining transcripts are shown in Fig. 5.

Induction with cellulose led to the early upregulation of 12 factors and downregulation of 16 TFs. Of these, the highest upregulation in expression was observed for FacB/Cat8/Sip4 (glucose-regulated transcriptional activators), Cre1/Mig1 (CCR regulator), Sut1/Pdr1 (negative/positive regulation of transcription by RNA polymerase II), and Pdr3 (positive regulation of transcription by RNA polymerase II). The other upregulated TFs were Rap1-Gcr1(ribosomal protein synthesis), Mcm1-Matα1 (cell cycle regulators), Rpn4 (proteasome regulator), Nit2 (activator of nitrogen-regulated genes) and Cbf1 (chromatin remodeling). The major down-regulated proteins were transcriptional regulators like Rox1, ToxE, Matα2, Reb1, Tbp and Xbp1; mediators of nutrient response like Gal4/Lac9, Lys14, Pho4 and Ume6; CCR regulator Sko1; the stress response regulator Mac1 and peroxisomal gene regulator Adr1.

## Discussion

Filamentous fungi, which usually grow on dead and decaying organic matter, rely on the secretion of a diverse arsenal of enzymes with varying specificities to achieve the degradation and mineralization of lignocellulosic biomass. These **C**arbohydrate **A**ctive en**Zymes** (CAZymes) include the cellulases (cellobiohydrolases, endoglucanases and BGLs), hemicellulases (chitinases, xylanases, mannanases, pectinases etc.) and other accessory enzymes (lignin peroxidases, oxidoreductases, lyases etc.). Research on the cellulase systems of various fungi and their modes of regulation has proved to be very useful for increasing the production of these enzymes in their native producers, and continue to be pursued in order to improve the enzyme titers and their activities.

For years, the ascomycete *Trichoderma reesei* and its mutants- Rut-C30, CL847 and QM9414- have been the preferred sources for industrial cellulase production. However, some of its drawbacks, such as the low content of BGLs which leads to product inhibition of the other cellulases, and enzyme intolerance to high glucose levels, have necessitated the need for external supplementation of the deficient proteins, which in turn drives up enzyme production costs. On the other hand, previous studies using PJ-1366 cellulases have established its excellent biomass hydrolyzing capability and superior secretion of CAZymes over *T. reesei* Rut-C30. Further characterization of the cellulase system of PJ-1366 requires identification of the proteins, both enzymes and regulators, that make up its lignocellulase system.

From the whole genome sequence data, 13 secreted cellulases were identified based on SignalP, dBCAN and UniProt annotations (Table 1). The number of exoglucanases (4), endoglucanases (7) and BGLs (2) are comparable to that of other fungi like *Trichoderma* and *Neurospora*. Therefore, the means for achieving higher cellulase activity is by massive upregulation and secretion of the cellulase genes, particularly of the cellobiohydrolases (Fig. 2 and Table 3). Induction levels and secretome abundance were lower for the BGLs compared to the CBHs and EGs, which implies that excellent catalytic activity and the low, but significant level of glucose tolerance (Fig. 3), can explain the higher level of BGL activity (compared to *T. reesei* RUT-C30) and low accumulation of the substrate cellobiose that was earlier reported by Sreeja-Raju et al.^4^. Product inhibition of the enzyme-catalyzed degradation of cellulose by non-complexed, fungal enzyme systems, have been described to be mediated by both competitive and non-competitive types of inhibition.^27^. Docking studies with cellobiose and glucose showed that both the PJ-1366 BGLs have different predicted binding sites for cellobiose and glucose, which can relieve competitive inhibition and contribute to their glucose tolerance (Supplementary File 2).

Other predicted properties are similar to those of general fungal cellulases. During hydrolysis of lignocellulose, the insoluble substrate is initially broken down at the solid–liquid interface via the synergistic action of endoglucanases and exoglucanases^28,29^. This generates soluble oligosaccharides, that are catalytically cleaved to glucose by the BGLs in the liquid phase. Therefore, BGLs do not generally possess Carbohydrate Binding Modules (CBMs) like the EGs and CBHs. Rather they encode domains like the Fn3 domain which are known to participate in maintaining structural stability of the enzyme and to ensure proper positioning of the substrate. The catalytic domains of BGLs are also larger (500–700 amino acids) than those of the other cellulases (200–300 amino acids), which contributes to the higher molecular weights of the BGLs (Supplementary File 1). The predicted pI of the cellulases varied from 4.25 to 7. For most proteins, the experimentally determined pI is very close to that predicted by online servers. However, it should be considered that cellulases tend to be post-translationally modified to ensure protein stability and activity, and in cases where the pI is observed to be shifted from the expected value, it is shown that this shift is often correlated to protein modifications^30^.

In order to conserve metabolic energy, fungal cellulases are expressed only in the presence of cellulosic polymers or other small molecule inducers. The expression is coordinately regulated at the transcriptional level, and is subject to repression when simple sugars like glucose are available for consumption (CCR). The regulatory pathways are triggered by the sensing of the aforementioned inducers, which are reported to be cellulose or hemicellulose-derived mono or oligosaccharides like sophorose, xylobioses or lactose. In *Penicillium*, gentiobiose (an α-1,6 linked disaccharide) has been reported as a potential inducer. The generation of these inducers is achieved by the low, constitutive expression of some cellulases, such as CbhII in *T. reesei*. The presence of inducers are sensed by regulatory proteins (like Clr proteins in *N. crassa*), which in turn induce the expression of permeases/transporters (like the cellodextrin transporter Cdt) for uptake of the substrate. Once internalized, the inducers drive the expression of other regulatory elements (receptors, kinases and DNA-binding proteins) which can positively affect cellulase expression. The regulation is also controlled by feedback loops, in which the upregulation of a downstream activator like Xyr1 can enhance expression of the upstream regulators like Clr1, leading to the massive transcription of cellulase genes. The expression is balanced by intracellular enzymes that regulate the inducers levels (like BglR), and protein trafficking and degradation regulators that can modulate cellulase expression in response to the secretory deficit in the cell.

In the case of PJ-1366, a combination of cellulose and wheat bran was found to be the best inducer of cellulase activity. Wheat bran alone did induce the expression of xylanases, BGLs and peroxidases, however, the presence of cellulose was required to drive cellulase and endoglucanase activity. Also, cellulose appeared to inhibit protease secretion compared to wheat bran, and this is a crucial advantage for ensuring stable cellulase activity.

Endoglucanases were strongly stimulated by the disaccharides cellobiose and lactose, however, the induction could not be sustained beyond the 3^rd^ day of incubation. The lack of significant extracellular BGL activity could be a reason for this, as the accumulation of the substrate (cellobiose) can inhibit EG activity. Another possibility is that once the available inducers are hydrolysed (by intracellular BGL action), there remains only glucose for consumption, which shifts the organism back to a CCR state. In any case, it is worthwhile to explore whether dosing the CW medium with additional lactose/cellobiose (either in batch/fed-batch mode) can lead to a sustained and increased expression of endoglucanases.

Secretome profiling at various time points in different media reiterated the high abundance of cellulases in the CW medium. Interestingly, there were only low levels of Cbh2 and Cbh3 in media with either cellulose or wheat bran alone, but the combination of cellulose and wheat bran led to high abundance of these two proteins, suggesting that, in addition to carbohydrates, the presence of other, possibly lignin-derived compounds are beneficial for triggering increased expression of cellulases. Eg4 is another protein which was induced for a short period in wheat bran, but had comparatively lower, albeit sustained expression in cellulose and CW medium. The expression of Eg6, on the other hand, seems to be negatively influenced by the presence of wheat bran, but strongly stimulated by lactose, and seems to be the protein responsible for the peak in CMCase activity observed on the 3^rd^ day in lactose-grown cultures.

BGLs were abundant in the wheat bran medium, in which six BGLs were detected, compared to 4 in the cellulose medium and only 2 in the CW medium. However, this abundance of BGLs in the WB medium did not translate to appreciably high enzyme activity, probably due to high protease activity of the culture (Fig. 1g). Since the abundance of BGL proteins in the CW secretome does not seem to correlate with the observed enzyme activity, it leads to 2 possibilities- (i) the observed high pNPGase activity in the CW medium might be due to the action of other glycanases (pNPGase assay is not strictly specific for BGLs, and may also be caused by the de-glycosylating action of other enzymes, including endoglucanases)^12^, or (ii) the two BGLs that are secreted in the CW medium have exceptionally high catalytic efficiency.

In addition to identifying the secreted cellulases of PJ-1366 and the substrates suitable for their expression, this study has also attempted to identify the early regulators of cellulase expression, based on the presence of binding sites of known TFs on the cellulase promoters, and correlating their distribution with the transcriptome and secretome profiles of the fungus. The major features of the complex cellulase regulatory network has been studied in many species of *Trichoderma*, *Neurospora* and *Aspergillus*, in which the master regulator of cellulase expression is the positive regulator Xyr1 or its orthologs (the deletion of *xyr1* abolishes cellulase expression on cellulose^31^). However, Xyr1 is only one of the last downstream effectors in the intricate control mechanism; as reviewed by Sukumaran et al.^4^ and others, the expression of xyr1 itself is regulated by CCR proteins like Cre1 and Ace1 via repression. Xyr1 requires another protein Ace2 for its functioning. The cellulase hyper-producer *T. reesei* Rut-C30 does not encode a functional Cre1- which is considered as one of the reasons for its superior cellulase production. On the other hand, another hyper cellulolytic strain- CL847- requires the expression of cre1 for the full induction of xyr1. Therefore, there exists significant differences in the regulatory pattern of cellulases among various genera and species.

In the case of PJ-1366, 704 TFs were differentially expressed on cellulose induction, of which 44 TFs were uniquely expressed in the presence of cellulose (Table 4). The highest fold change in expression was observed for a sugar transporter STL1, which encoded both transporter and DNA-binding domains (Supplementary File 4). There have been previous reports of transporters with transceptor activities- the cellodextrin transporters CDT-1 and CDT-2 in *N. crassa*^32^, and the cellobiose transporter CRT1 in *T. reesei*^33^ participate in signaling pathways that result in downstream activation of cellulase gene expression. In the case of the PJ-1366 transporter, it had a XylE superfamily domain, which is a Major Facilitator Superfamily of transporter proteins that participate in the proton-coupled uptake of D-xylose against its concentration gradient^34,35^, and is competitively inhibited by glucose^36^.

Further, it was seen that the abundance of cellulases in the secretome was positively correlated with the TF-binding sites of 13 factors. Among these, 5 genes- *xyr1*, *ace2*, *cre1*, *pdr3* and *gcn4*- were upregulated on cellulose induction (Table 5). Xyr1, a Zn2Cys6 type transcription factor, and its orthologues like XlnR, are established master regulators of cellulase expression. Xyr1 directly influences the expression of xylanases and cellulases as well as the transcription of inducer-providing enzymes like β -xylosidases in *T. reesei* and *A. niger*^37^. Ace2 is also an enhancer, and binds upstream of Xyr1 to drive the expression of genes under its control. Similar to Xyr1, Gcn4 is also a master transcriptional regulator that mediates the cellular response to amino acid starvation^38^. It activates transcription by recruiting multiple coactivators, including the mediator complex, the SAGA complex, and the SWI/SNF complex, to enable assembly of the pre-initiation complex at core promoters. The upregulation of *gcn4*/*cpc1* is indicative of linkages between carbon and nitrogen metabolism in the organism. One of the known cellular responses to glucose starvation is deamination of amino acids, which can then enter the pathways of glucose metabolism as pyruvate, acetyl CoA, or several components of the citric acid cycle. This leads to amino acid starvation that triggers the expression of *gcn4*. Based on the transcript levels, it may be hypothesized that Gcn4 works along with other TFs to increase cellulase expression needed to restore glucose levels.

The shift from glucose to cellulose metabolism represents a stressed state for the organism, both with regard to nutrient starvation and the associated changes in protein translation and secretion levels that are needed to restore homeostasis. Therefore, it is natural that stress-responsive pathways and genes are upregulated in this scenario. In yeasts, the Pdr (Pleiotropic Drug Resistance) proteins Pdr1 and Pdr3 regulate the expression of a multitude of genes, including those encoding ABC transporters, phospholipid biosynthetic enzymes, transmembrane domain-containing proteins, oxidoreductases, sphingolipid biosynthetic enzymes, proteins involved in DNA repair, and other transcription factors like Rpn4. In short, they participate in controlling membrane biogenesis by adjusting the production of different membrane proteins^39^. The role of Pdr orthologues is not defined in filamentous fungi, and is therefore an exciting avenue for exploration.

An orthologue of Cre1 was upregulated on cellulose induction in PJ-1366. Cre1 is reported as the central regulator of CCR in many filamentous fungi^40^, and is therefore expected to be downregulated on glucose depletion, contrary to the effects observed here. According to the studies of Portnoy et al.^41^, Cre1 has functions other than the regulation of CCR, for example, in the presence of an inducer like lactose, a functional Cre1 is required to drive the expressions of both *xyr1* and *ace2*. A positive effect of the *A. nidulans* CreA on gene expression has also been reported previously^42^, and it may be postulated that similar mechanisms are at play here.

Finally, 28 differentially expressed TFs were detected which had binding sites on the promoters of the aforementioned set of primary cellulase regulators. Since these factors are upstream of the immediate cellulase regulators, they may be considered as early modulators of cellulase expression in PJ-1366. Of these, 12 were upregulated and 16 were downregulated in response to cellulose induction (Fig. 5). The highest positive fold change was observed for FacB and the CreA-like Mig1 protein. FacB, and its orthologues like Cat8 and Sip4, have been previously implicated in de-repression of a variety of yeast genes under non-fermentative growth conditions, via binding to carbon source-responsive elements (CSRE) in the promoters of genes under their control. In both Cat8 and Sip4, activation requires phosphorylation, much like Ace2 in *T. reesei*^43^. FacB is reported as a regulator of acetamidase and the acetate utilization enzymes acetyl-CoA synthase, isocitrate lyase and malate synthase.

The other upregulated TFs included the regulators of transcription by RNA polymerase II- Sut1/Pdr1 and Pdr3; Cbf1, which is a modulator of chromatin accessibility; regulators controlling the protein synthesis and degradation pathways like Rap1, Gcr1 and Rpn4; the cell cycle regulators Mcm1 and Matα1; and yet another TF participating in the activation of nitrogen-regulated genes- Nit2. The effective production and secretion of proteins requires a robust secretion pathway, i.e., a well-coordinated machinery for protein synthesis, translation, translocation, folding, modification and transport to the cell exterior. The deregulation of CCR leads to massive transcription of cellulases and their regulators. Fungi respond to accumulation of polypeptides in the ER by activating the UPR pathway (chaperones, foldases, proteasomes and glycosylation machineries). The Rap1 protein has dual regulatory roles- it activates most ribosomal protein genes; but can also repress these genes in response to a secretory defect. Mcm1, in addition to being a cell cycle regulator, also participates in the control of synthesis of cell wall/membrane structures, and regulation of heat-shock-inducible glycoprotein production. Secretion stress also induces *cpc1* in *T. reesei*^44^, which, along with Gcn4 and Nit2, is implicated in the regulation of amino acid synthesis. The orthologues of Nit2- Are1 in *Trichoderma* and AreA in *Aspergillus*, have been reported to positively modulate both cellulase expression and secretion of proteases^45^, which can be interpreted as cellular responses to the perceived nutrient deficit.

The highest decrease in fold change was seen for Rox1, which is known as a repressor of transcription from RNA polymerase II promoter in response to stress. In agreement with previous reports, Ste12- which regulates transcription, pseudohyphal growth and sexual reproduction in fungi, and is an inhibitor of cellulase, chitinase and protease formation via modulation of MAP kinase signaling pathways^46–48^, was downregulated on cellulose induction. Regulators of nutrient metabolism like Gal4/Lac9, Lys14, Pho4, Ume6 and Sko1 were also downregulated. Among these, Pho4, which is a positive regulator of phosphate metabolic processes in response to phosphate starvation, also participates in chromatin remodeling. Sko1 is a CCR modulator that has positive functional interactions with Mig1. Since the analysis presented here is of the early transcriptional environment following cellulose induction, it may be reasoned that the downregulation of Sko1 probably leads to gradual decrease in the levels of the master CCR effector Cre1 (which is an orthologue of Mig1).

Stress-response modulators that were downregulated included Reb1, Xbp1 and Mac1. Two of these proteins- Xbp1 and Reb1- are associated with translation and secretion. Xbp1, which has been more widely characterized in mammalian cells than fungal cells, is a regulator of the ER-associated degradation (ERAD) pathway^49^ which is activated on the aggregation of proteins in the lumen of the ER. Reb1 controls translation rates by modulating the termination of RNA polymerase I catalyzed transcription of rRNA genes^50^.

Mac1 is involved in Cu/Fe utilization and stress resistance. It also regulates the transcription of sugar transporters like Ctr1 and Ctr3 via copper ion responsive elements in their promoters. Further, Mac1 is also required for degradation of Ctr1, which is a permease that plays an essential role in transmitting the cellulase gene-inducing signal independent of its transport functions^51^. The *adr1* gene, which was also downregulated on induction, encodes a peroxisomal gene regulator. Peroxisomes are now recognized as important signaling organelles, capable of generating signaling molecules^52,53^, and also integrate external signals to trigger specific cell developmental responses^54^. Furthermore, peroxisomes also function as a scaffold for the assembly of specific macromolecular signaling complexes, which participate in the orchestration of complex signaling networks^55–57^. More recently, Mattilda et al.^58^ demonstrated that Adr1 is essential for the hypoxia-driven expression of CAZymes in the white rot fungus *Phlebia radiata*. Other studies have also demonstrated that Adr1 coordinates the biochemical pathways that generate acetyl-CoA and NADH from non-fermentable substrates,^59^ and so it is agreeable that aerobic culture conditions do not significantly affect the levels of Adr1 in PJ-1366.

While a broad overview of the changes in the transcriptional landscape in response to cellulose induction can be gauged from the above data, it is cautioned that, as can be seen from Supplementary File 6, the percentage identity is quite low (varying from 27 to 60% for most proteins) among the characterized TFs and their corresponding orthologues in PJ-1366. Also, there is more to TF–DNA binding than primary nucleotide sequence preferences. Accumulating evidence supports the widespread contributions of factors like flanking sequences and DNA shape in modulating sequence recognition. Interacting cofactors and TFs can also alter sequence preference^60^. Therefore, such additional features, together with differential TF expression, are the ultimate determinants of substrate-specific TF binding.

## Conclusion

The lignocellulose-degrading enzyme system of *Penicillium janthinellum* NCIM-1366 consists of 4 cellobiohydrolases, 7 endoglucanases and 2 BGLs, which is larger and more diverse than that of the industrial standard for cellulase production- *Trichoderma reesei* Rut-C30. All the cellulases are differentially expressed in response to induction. A combination of wheat bran and cellulose was identified as the best inducer of cellulase expression and protein secretion in PJ-1366. The xylose transporter XyrE was exclusively expressed in the presence of cellulose, and might be responsible for the subsequent upregulation of the cellulase master regulator Xyr1. By correlating transcriptome and secretome profiles with the distribution of transcription factor-binding sites, it was seen that induction with cellulose led to the differential expression of factors regulating transcription, translation, secretion, nutrient metabolism and stress responses, indicating that these pathways are co-regulated with the expression of cellulase genes in this fungus. Since cellulase production by fungi is tightly regulated in response to environmental cues, this knowledge base is a crucial pre-requisite in efforts to improve cellulase secretion via modifying culture conditions and targeted genetic interventions.

## Methods

### Strain

The strain *Penicillium janthinellum* NCIM 1366 was obtained from Dr. D. V. Gokhale at National Chemical Laboratory, Pune, India. The strain was generated by mutating the wild-type strain *P. janthinellum* NCIM 1171 using Ethyl Methyl Sulfonate, followed by UV irradiation^61^.

### Identification of secreted cellulases

Proteins were predicted from the NGS data of PJ-1366 using AUGUSTUS^62^, and their secretory nature was determined using SignalP 5.0.^63^. From the proteins with predicted signal peptides, CAZymes were identified using the dBCAN server^64^. Proteins identified as extracellular CAZymes were annotated using the UniProt Fungi database.

### Physicochemical properties and domain analysis of the cellulases

The physicochemical properties of the cellulases- length, molecular weight, pI and hydrophobicity- were predicted using the Sequence Manipulation Suite^65^. The distribution of known protein domains was analysed using NCBI’s Conserved Domain Database^66^.

### Culture conditions

PJ-1366 was grown on Potato Dextrose Agar (PDA) slants for 15 days (or until the complete appearance of grayish spores) at 30 °C. For enzyme production, the spores were transferred to Mandels and Weber medium^67^, pH 5.5, to attain a concentration of 1 × 10^5^ spores/mL. The substrate concentration was 1% w/v, and the cultures were incubated at 30 °C, 200 rpm for varying time periods.

### Enzyme activities

The fungal biomass was separated by filtration, and the filtrates were further clarified by centrifugation at 8000 g, 4 °C for 20 min. Secreted proteins in the culture were detected by a modified Bradford’s method^68,69^. Standards were prepared using 0.2–2 mg/mL of BSA solution.

Cellulase activities of the supernatants were determined according to the protocols described by Ghose et al.^70^. Total cellulase (FPase), endoglucanase (CMCase) and xylanase activities were quantified in terms of reducing sugar (glucose/xylose) released using the DNS assay^71^. BGL activity was quantified in terms of micromoles of pNPG released under standard assay conditions. To determine glucose tolerance of the BGLs, citrate–phosphate buffer (50 mM, pH 4.8) containing varying concentrations of glucose was used in the assay.

Peroxidase activity (an indicator of ligninolytic action) was determined using the *Peroxidase Activity Assay Kit* (MAK092) from Sigma-Aldrich, as per the manufacturer’s protocol for colorimetric tests. Protease activity was determined using the *Pierce™ Colorimetric Protease Assay Kit* (Catalog number: 23263) from ThermoFisher Scientific, according to the manufacturer’s protocol.

The results of enzyme assays presented in Figs. 1 and 3 are the average of biological triplicates.

### Homology modelling and docking studies

Homology modelling of PJ-1366 BGLs was done using the SWISS-MODEL server^72^. The structures of cellobiose (CHEBI:**17,057**), gentiobiose (CHEBI:**28,066**) and glucose (CHEBI:**17,234**) were obtained from the ChEBI database^73^. Docking was performed using PatchDock^74^, binding energies were determined using FireDock^75^, and the docked structures were visualized using EzMol 2.1^76^.

### Transcriptome profiling

PJ-1366 was grown on Mandels and Weber medium containing 1% w/v glucose as the carbon source. After 48 h, the biomass was separated by filtration, washed with sterile, nuclease-free water, and transferred to fresh medium containing either 1% w/v glucose (control) or cellulose (test). Four hours post induction, the biomass was again filtered out, washed and flash-frozen in liquid nitrogen. RNA isolation, transcriptome sequencing, de novo assembly and annotation was carried out at OmicsGen LifeSciences Pvt. Ltd., Kochi, Kerala India.

Gene expression was calculated using RSEM v0.6.1^77^, which measures gene expression in FPKM (Fragment per Kilo bases per Million reads). FPKM was calculated as:

1 $$FPKM = 10^{9} {\text{C}}/NL$$

where ***C*** is the number of fragments that uniquely aligns to the gene of interest, ***N*** is the total number of fragments that uniquely aligns to the reference genome, and ***L*** is the base length of the gene of interest.

### Secretome profiling

Secretome profiling of the culture supernatants was done at 0, 3, 7 and 13 days post inoculation. After separation of the fungal biomass from 100 ml cultures, the supernatants were lyophilized and re-dissolved in 1 ml of 50 mM citrate–phosphate buffer, pH 4.8. Proteins were precipitated using the acetone-TCA method, as per the protocol of Niu et al.^78^. The precipitated proteins were redissolved in ammonium bicarbonate buffer (pH 7, 50 mM) and concentrations were normalized.

The samples were digested using Trypsin^79^, and proteome profiling was performed in duplicates by liquid chromatography tandem mass spectrometry (LC–MS/MS) at the Mass Spectrometry & Proteomics Core facility of Rajiv Gandhi Centre for Biotechnology, Trivandrum, India. The LC–MS/MS acquired raw data were analyzed by Progenesis QI for Proteomics v3.0 (NonLinear Dynamics, Waters, UK) for protein identification using the protein database of *Penicillium* downloaded from UniProt repository, as well as a user-provided database of predicted PJ-1366 proteins. The results presented are the mean of duplicates.

### Promoter identification and distribution of TF-binding sites

Promoters were identified from the intergenic space between two successive ORFs from the AUGUSTUS protein prediction data, with the maximum length arbitrarily set to 1500 bp upstream of the ORF. Binding sites of known fungal transcription factors were determined using the PROMO server^80^ (species- ascomycetes). Information on the TFs were obtained from the TRANSFAC^81^ database, and pathway mapping was done using the KEGG server^82–84^. Gene ontology predictions were done using the PANTHER database^85^.

### Correlation analysis

Correlation analysis was performed using the Analysis ToolPak statistical analysis package which is available in Excel for Microsoft 365^86^. Correlations were determined for the distribution of TF binding sites on the promoters of cellulases and the protein abundance on 7 day old cultures grown on 1% cellulose-wheat bran mixture as the carbon source.

## Supplementary Information

Supplementary Information.

## Data Availability

The whole genome sequence of PJ-1366 is available on GenBank with accession number **GCA_002369805.1**. The contig level assembly is present under accession number **ASM236980v1.** All essential data generated or analyzed during this study are included in this published article and its supplementary information files. More elaborate datasets generated during and/or analyzed during the current study are available from the corresponding author on reasonable request.
