# Supplementary material for: Early cellular events and potential regulators of cellulase induction in Penicillium janthinellum NCIM 1366
Source: Sci Rep. 2023 Mar 28;13:5057. doi: 10.1038/s41598-023-32340-x (PMC10050438; doi:10.1038/s41598-023-32340-x)
Supplement: Supplementary file 1 — Supplementary Information. [file 41598_2023_32340_MOESM1_ESM.pdf]

# Early cellular events and potential regulators of cellulase induction in *Penicillium janthinellum* NCIM 1366

Meera Christopher, AthiraRaj Sreeja-Raju, Amith Abraham, Digambar Vitthal Gokhale, Ashok Pandey, and Rajeev K Sukumaran

## Supplementary Files

**Supplementary File 1**. Domain distribution on PJ-1366 cellulases

**> CBH1**

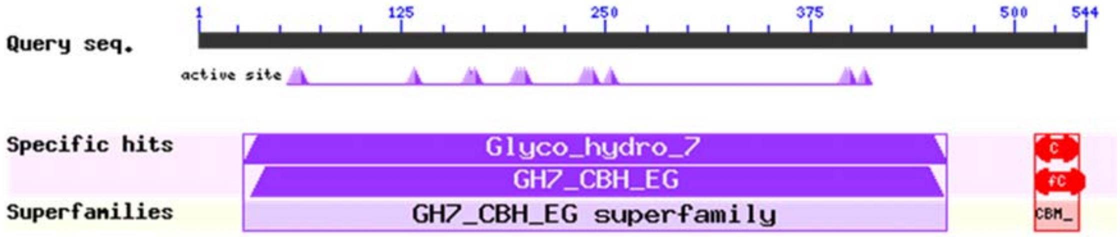


**> CBH2**

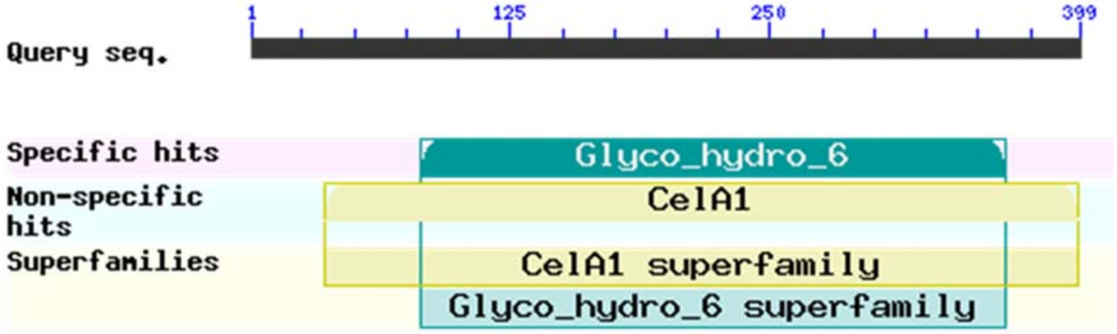


**> CBH3**

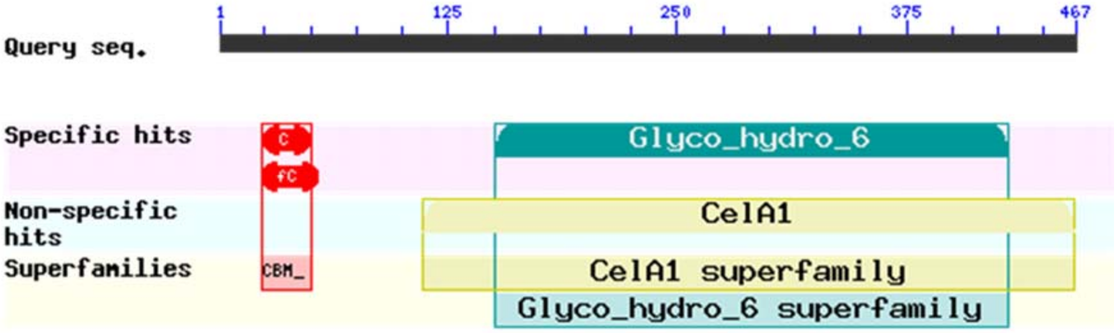

**> CBH4**

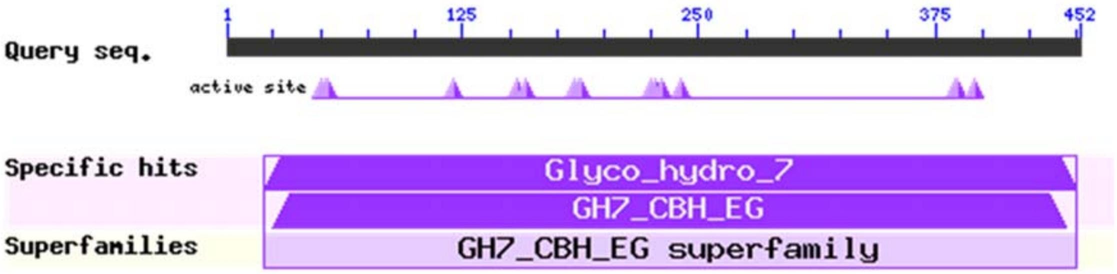


**> EG1**

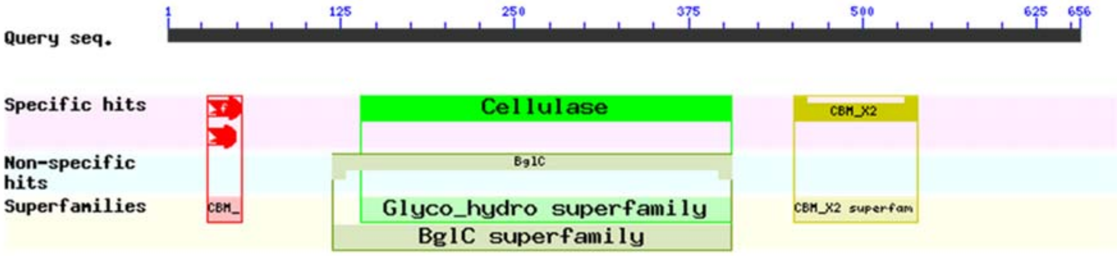


**>EG2**

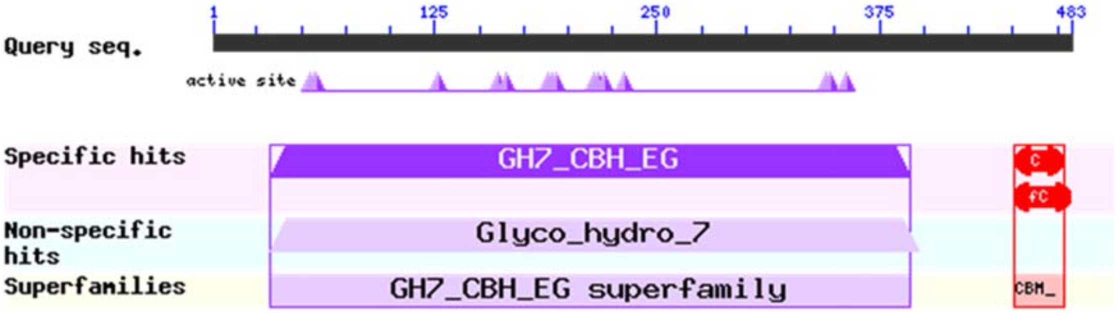


**> EG3**

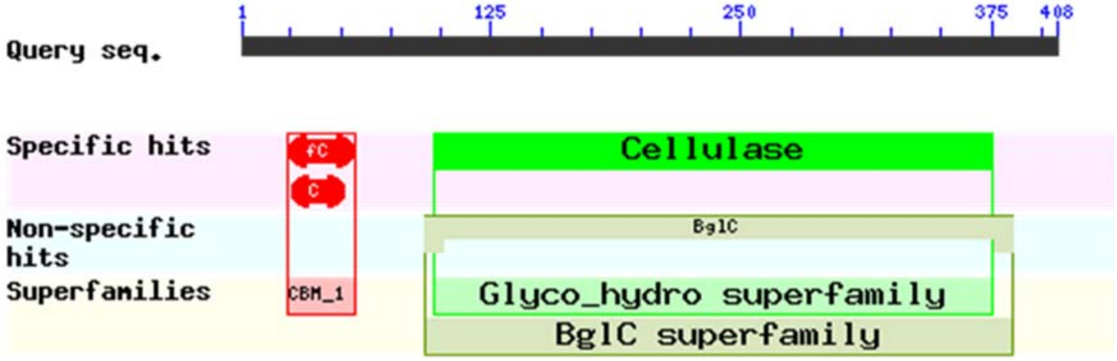

> EG4

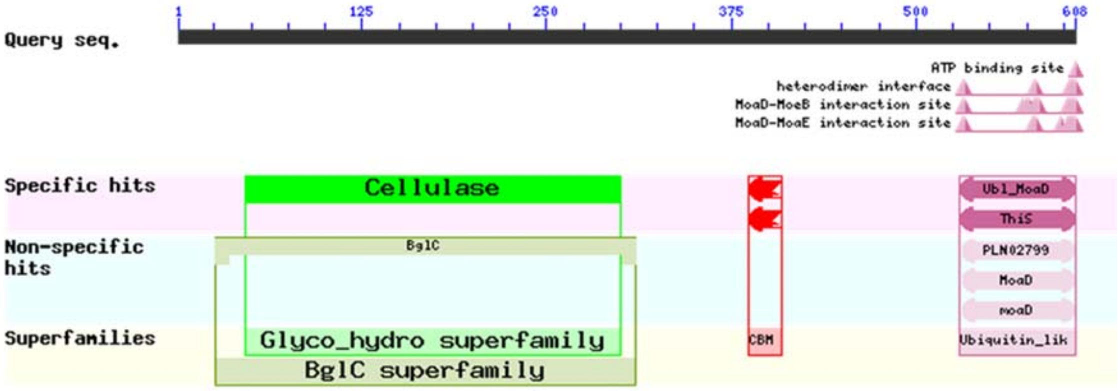


> EG5

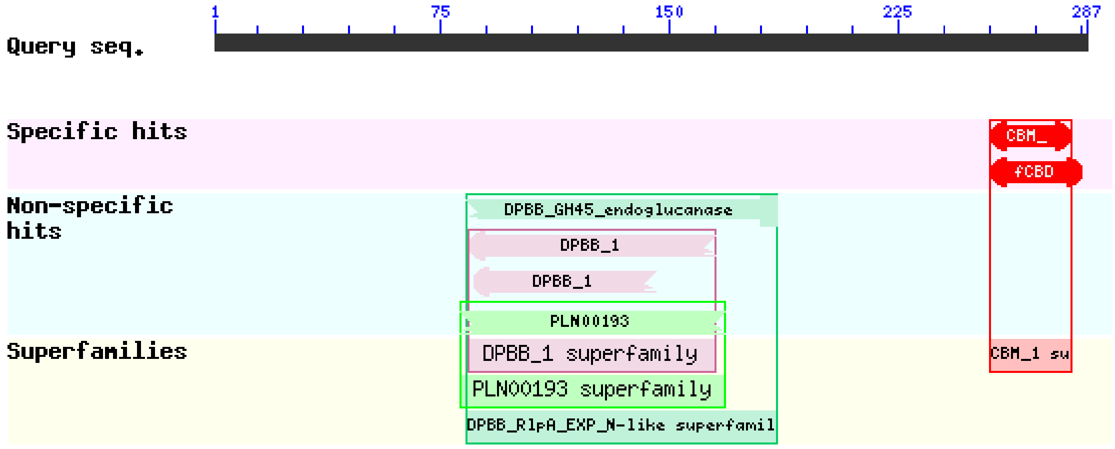


> EG6

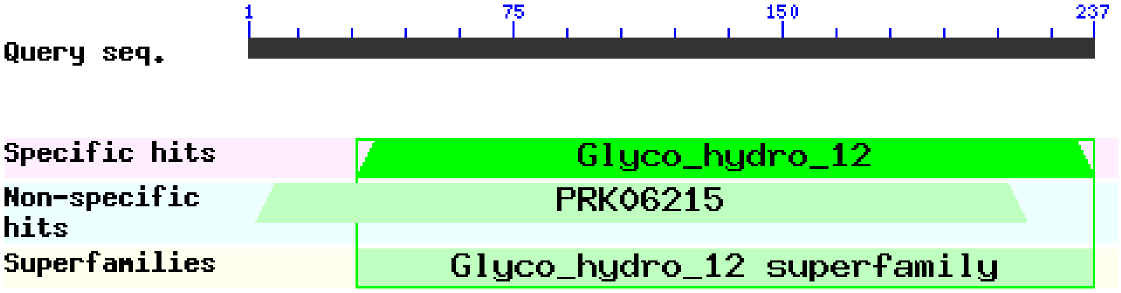


>EG7

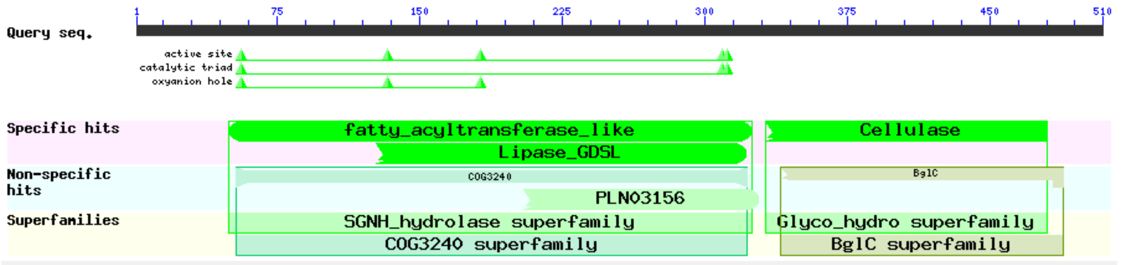

**>BGL1**

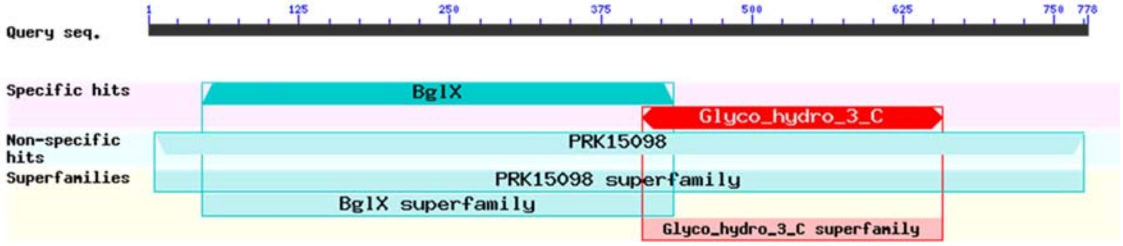
Query seq.
1
125
250
375
500
625
750
778
Specific hits
BglX
Glyco_hydro_3_C
Non-specific hits
PRK15098
Superfamilies
PRK15098 superfamily
BglX superfamily
Glyco_hydro_3_C superfamily

**> BGL2**

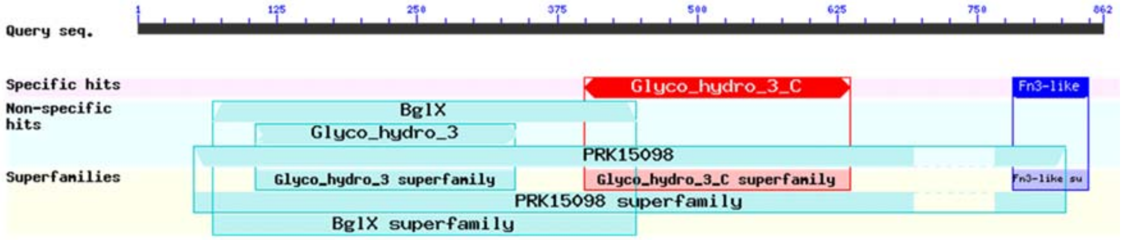
Query seq.
1
125
250
375
500
625
750
862
Specific hits
Glyco_hydro_3_C
Fn3-like
Non-specific hits
BglX
Glyco_hydro_3
PRK15098
Superfamilies
Glyco_hydro_3 superfamily
Glyco_hydro_3_C superfamily
Fn3-like su
PRK15098 superfamily
BglX superfamily

**Supplementary File 2.** Docking of PJ-1366 BGLs with **a.** Cellobiose (substrate); **b.** Gentiobiose (potential inducer), and; **c.** Glucose (product). BGL1 does not bind glucose in the same site as cellobiose, while the affinity of glucose for the cellobiose-binding site in BGL2 is -6.15 kcal/mol

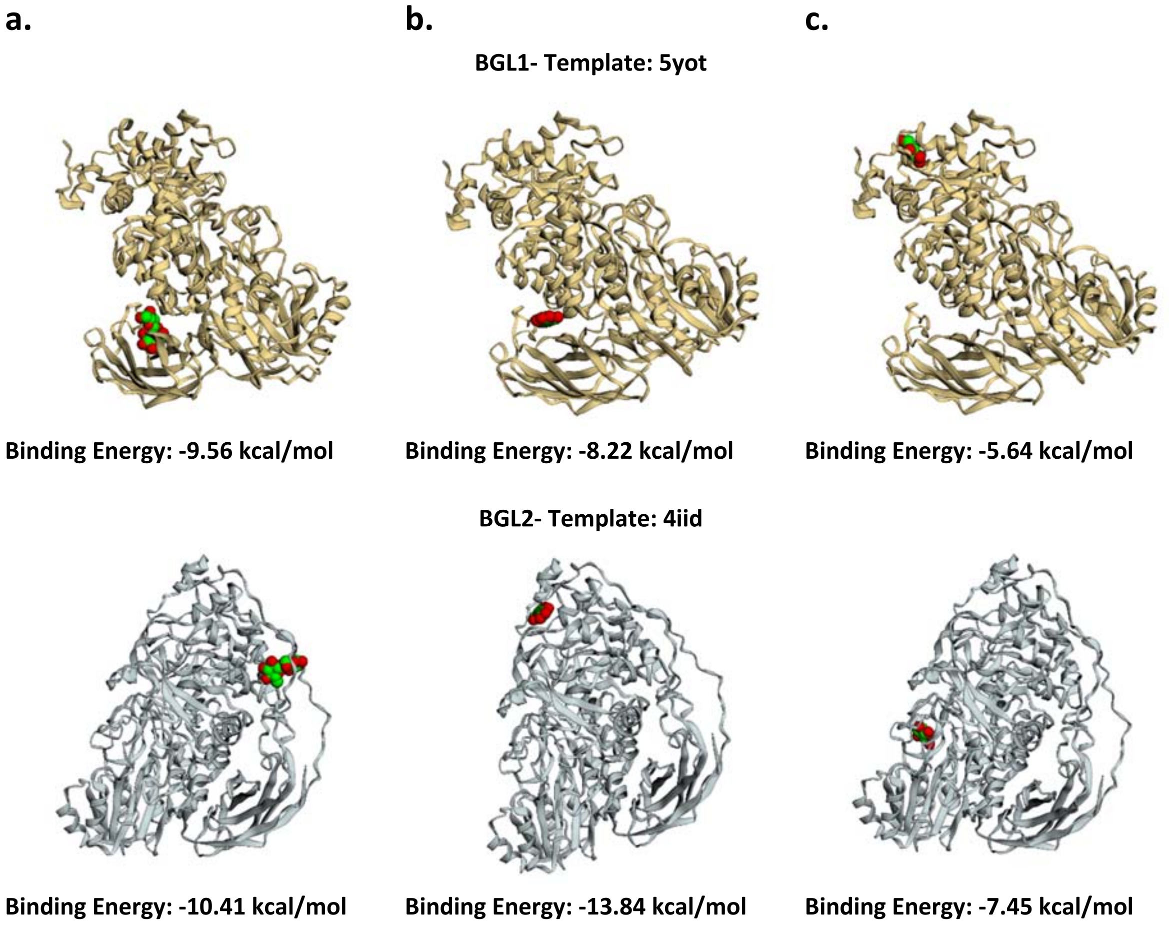

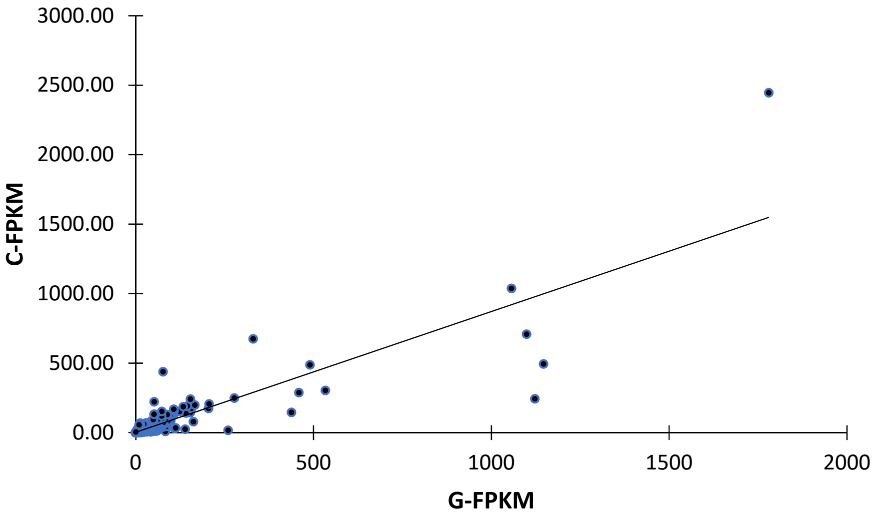


**Supplementary File 3.** Comparison of the expression levels of TF transcripts on cellulose (y-axis) vs glucose (x-axis)

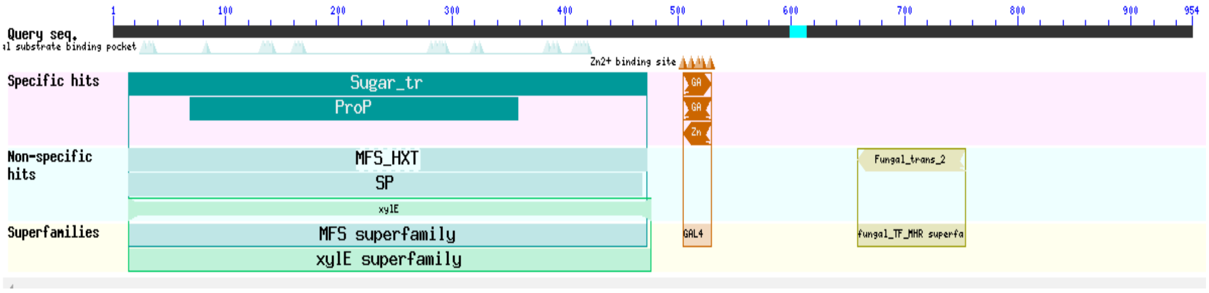


**Supplementary File 4.** Domain analysis of ctg7180000015228.g98

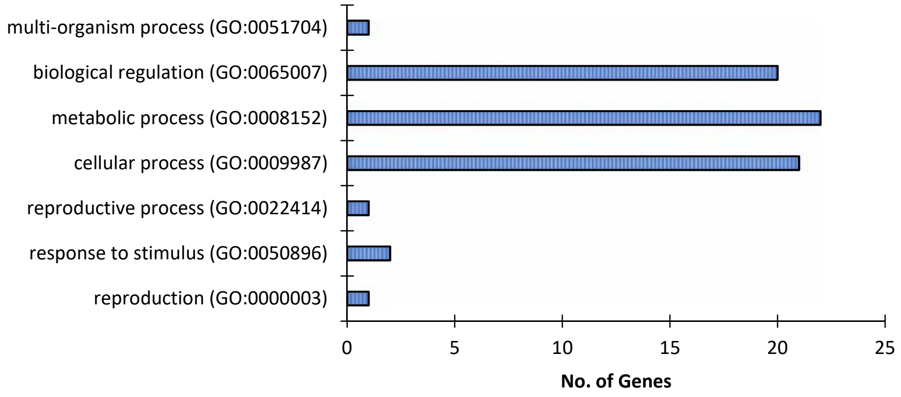


**Supplementary File 5.** Ontology analysis of TFs with binding sites on cellulase promoters
*32 of the 46 TFs were mapped onto different biological processes*

**Supplementary File 6.** Mapping of predicted early cellulase regulators to the PJ-1366 genome and transcriptome

| Factor | UniProt ID | PJ Protein | % Identity | Transcript ID | % Identity2 | G_FPKM | C_FPKM |
|---|---|---|---|---|---|---|---|
| FACB | O13461 | | | | | | |
| CAT8 | P39113 | ctg7180000015115.g238 | 60.85 | TRINITY_DN10653_c0_g1_i1 | 100 | 33.45 | 112.36 |
| SIP4 | P46954 | | | | | | |
| PDR3 | P33200 | ctg7180000014579.g118 | 26.11 | TRINITY_DN11674_c0_g1_i1 | 100 | 1.69 | 3.7 |
| ROX1 | P25042 | ctg7180000015059.g151 | 46.43 | TRINITY_DN11920_c0_g1_i1 | 100 | 10.8 | 3.76 |
| MATa2 | P0CY08 | ctg7180000014600.g260 | 34.18 | TRINITY_DN12586_c0_g1_i1 | 100 | 4.5 | 4 |
| PHO4 | P07270 | ctg7180000014400.g249 | 34.95 | TRINITY_DN12941_c0_g1_i1 | 100 | 54.2 | 38.86 |
| GCN4 | P03069 | ctg7180000009961.g293 | 29.11 | TRINITY_DN1536_c0_g1_i1 | 95.662 | 707.67 | 1099.07 |
| CPC1 | P11115 | | | | | | |
| TOXE | O74205 | ctg7180000009916.g158 | 29.14 | TRINITY_DN16568_c0_g1_i1 | 100 | 32.46 | 17.14 |
| RAP1 | P11938 | ctg7180000014810.g152 | 36.36 | TRINITY_DN16690_c0_g1_i1 | 100 | 2.36 | 3.04 |
| MIG1 | P27705 | ctg7180000010025.g78 | 52.63 | TRINITY_DN18772_c0_g1_i1 | 100 | 144.93 | 438.18 |
| UME6 | P39001 | ctg7180000015101.g196 | 36.36 | TRINITY_DN2081_c0_g1_i1 | 100 | 12.63 | 6.43 |
| SUT1 | P53032 | ctg7180000015379.g171 | 58.33 | TRINITY_DN21703_c0_g1_i1 | 100 | 11.74 | 25.63 |
| PDR1 | P12383 | | | | | | |
| MCM1 | P11746 | ctg7180000014443.g45 | 74.74 | TRINITY_DN21880_c0_g1_i1 | 100 | 88.73 | 98.18 |
| XBP1 | P40489 | ctg7180000009974.g110 | 46.67 | TRINITY_DN23140_c0_g1_i1 | 100 | 4.17 | 4.01 |
| RPN4 | Q03465 | ctg7180000015229.g190 | 44.3 | TRINITY_DN24330_c0_g1_i1 | 96.779 | 303.23 | 533.27 |
| MAC1 | P35192 | ctg7180000014444.g93 | 47.37 | TRINITY_DN25511_c0_g1_i1 | 100 | 159.47 | 136.41 |
| NIT2 | P19212 | ctg7180000014579.g108 | 39.95 | TRINITY_DN3063_c0_g3_i1 | 99.658 | 18.09 | 34.35 |
| GAL4 | P04386 | ctg7180000015120.g14 | 29.19 | TRINITY_DN4403_c0_g2_i1 | 100 | 33.61 | 31.89 |
| LAC9 | P08657 | | | | | | |
| ADR1 | P07248 | ctg7180000014442.g38 | 50 | TRINITY_DN6309_c0_g1_i1 | 98.739 | 5.42 | 4.72 |
| SKO1 | Q02100 | ctg7180000015244.g235 | 35.37 | TRINITY_DN6578_c0_g1_i2 | 100 | 10.18 | 6.54 |
| CBF1 | P17106 | ctg7180000015126.g146 | 35.1 | TRINITY_DN7135_c0_g2_i1 | 99.202 | 8.82 | 14.48 |
| STE12 | P13574 | ctg7180000015092.g141 | 60 | TRINITY_DN7299_c0_g2_i1 | 100 | 62.98 | 52.79 |
| REB1 | P21538 | ctg7180000015100.g153 | 39.82 | TRINITY_DN7500_c0_g1_i1 | 29.577 | 0.04 | 0.03 |
| LYS14 | P40971 | ctg7180000009927.g33 | 37.84 | TRINITY_DN7740_c0_g1_i1 | 100 | 12.02 | 10.19 |
| TBP | P13393 | ctg7180000014397.g136 | 91.11 | TRINITY_DN8049_c0_g1_i1 | 100 | 258.81 | 208.91 |
| MATa1 | P0CY11 | ctg7180000015122.g49 | 30 | TRINITY_DN8704_c0_g1_i1 | 100 | 15.01 | 15.93 |
| SWI4 | P25302 | ctg7180000010135.g36 | 39.46 | TRINITY_DN9577_c1_g1_i1 | 100 | 13.98 | 9.11 |
| SWI6 | P09959 | ctg7180000015097.g76 | 27.49 | TRINITY_DN9601_c0_g1_i1 | 100 | 10.12 | 8.41 |
| GCR1 | P07261 | ctg7180000014753.g103 | 33.33 | TRINITY_DN9776_c0_g1_i2 | 99.704 | 14.81 | 17.71 |
